# Anesthetic management of occult traumatic pneumothorax with one-lung ventilation avoiding prophylactic chest tube: a case report

**DOI:** 10.1186/s40981-025-00812-w

**Published:** 2025-08-29

**Authors:** Kazuyasu Aoki, Taku Mayahara, Tomohiro Katayama, Yuya Hirai, Masao Uchihashi, Ryosuke Fukuoka

**Affiliations:** 1 Department of Emergency and General Medicine, Kobe Ekisaikai Hospital, 1-21-1 Manabigaoka, Kobe, Tarumi-ku, Hyogo 655-0004 Japan; 2 Department of Anesthesiology, Kobe Ekisaikai Hospital, Kobe, Japan

**Keywords:** Traumatic pneumothorax, Occult pneumothorax, One-lung ventilation, Positive pressure ventilation, general anesthesia

## Abstract

**Background:**

Guidelines recommend prophylactic chest tube placement in patients with traumatic pneumothorax who require positive pressure ventilation to prevent tension pneumothorax. However, chest tube insertion is not without complications, and avoiding it when safely possible is desirable.

**Case presentation:**

A man in his 50 s with a left clavicle fracture and mild left-sided occult pneumothorax on computed tomography was scheduled for surgery under general anesthesia. Conservative management was chosen given the absence of respiratory symptoms and stable imaging. To minimize the risk of pneumothorax progression during positive pressure ventilation, one-lung ventilation was employed to avoid ventilating the affected lung. Surgery proceeded uneventfully, with transient hypoxemia that was managed by increasing FiO_2_ to 100%. Postoperative imaging confirmed re-expansion of the left lung and no pneumothorax progression. The patient was discharged in good condition.

**Conclusions:**

General anesthesia was safely managed without prophylactic chest tube placement by employing one-lung ventilation in a patient with occult traumatic pneumothorax.

## Background

Traumatic pneumothorax is a common complication of blunt chest trauma. When positive pressure ventilation (PPV) is anticipated—such as during general anesthesia—trauma guidelines generally recommend prophylactic chest tube insertion to prevent the development of tension pneumothorax [1, 2]. Consequently, even patients initially managed conservatively are often subjected to chest tube insertion when surgery under general anesthesia with PPV is planned [3]. However, chest tube insertion is not without complications, including infection, pain, or lung injury [4, 5], and avoiding unnecessary chest tube placement is desirable whenever possible.

With the increasing use of computed tomography (CT), minor pneumothoraces that are not visible on plain radiographs—referred to as occult pneumothoraces (OPTX)—are now being detected more frequently [5]. This report describes a case of clavicle fracture repair in a patient with OPTX managed under general anesthesia. One-lung ventilation (OLV) was employed to avoid applying PPV to the affected lung, successfully allowing surgery to proceed without prophylactic chest tube placement. To our knowledge, no previous case reports have described the use of OLV during general anesthesia specifically to avoid prophylactic chest tube placement in patients with traumatic pneumothorax. This case suggests a potential approach to anesthetic management in such patients.

## Case presentation

**Fig. 1:**
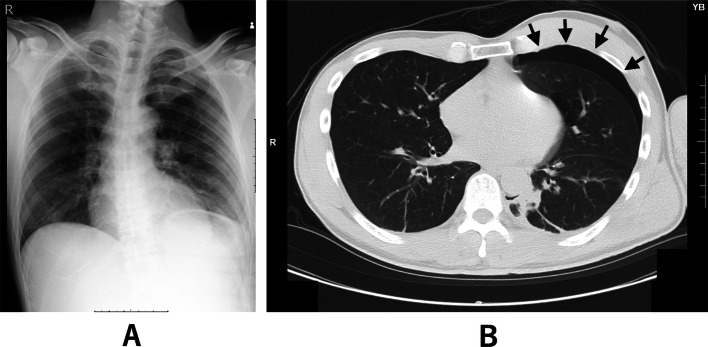
Chest radiography (**A**) showed no obvious pneumothorax, while computed tomography (**B**) revealed a mild left-sided pneumothorax

A man in his 50 s with no significant medical history (height 177 cm, weight 73 kg) presented to the emergency department with left shoulder pain after a bicycle fall. Imaging revealed fractures of the left clavicle and the left second and third ribs. While the chest radiograph did not show any obvious pneumothorax (Fig. 1A), CT demonstrated a mild left-sided pneumothorax (Fig. 1B). There was no significant hemothorax, pulmonary contusion, or subcutaneous emphysema. Given the absence of respiratory symptoms, a conservative approach without chest tube placement was adopted. Follow-up chest radiography and CT the next day confirmed that there was no progression of the pneumothorax. Surgical fixation of the clavicle fracture was scheduled for post-injury day 6.

On the morning of surgery, preoperative chest radiography showed no visible pneumothorax, consistent with the findings on the previous radiograph. General anesthesia with tracheal intubation and PPV was chosen; however, because the pneumothorax cavity was narrow, chest tube insertion was considered to carry a relatively high risk of lung injury. Therefore, prophylactic chest tube placement was avoided, with plans for emergency drainage if pneumothorax worsened intraoperatively. To further reduce the risk of exacerbation, OLV was chosen as a precautionary measure to minimize positive pressure exposure to the affected lung.

**Fig. 2:**
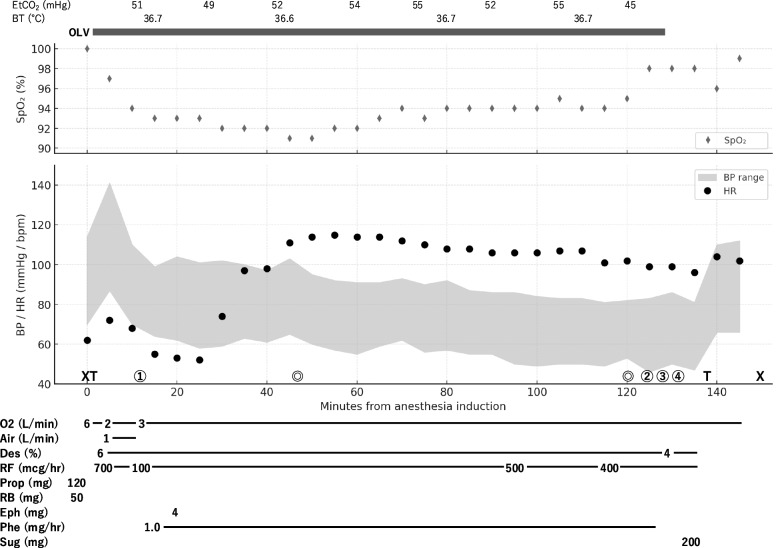
Digitized anesthesia chart. SpO₂: peripheral oxygen saturation; EtCO₂: end-tidal carbon dioxide; BP: blood pressure; HR: heart rate; BT: body temperature; Des: desflurane; RF: remifentanil; Prop: propofol; RB: rocuronium bromide; Eph: ephedrine; Phe: phenylephrine; Sug: sugammadex. ✖ indicates the start and end of anesthesia; T indicates intubation and extubation; ⊚ indicates the start and end of surgery; ① indicates the change to the beach-chair position; ② indicates repositioning to the supine position; ③ and ④ correspond to the timing of chest radiography shown in Fig. 3A and B, respectively

General anesthesia was induced with 120 mg of propofol, 700 µg/h of remifentanil, and 50 mg of rocuronium. After two or three manual mask ventilations, a 37 Fr left-sided double-lumen endotracheal tube was placed, and one-lung ventilation of the right lung was initiated. Pressure-controlled ventilation with volume guarantee was used (tidal volume 300–400 mL; respiratory rate 12–15 breaths per minute; positive end-expiratory pressure 6 cmH₂O). Initially, ventilation was performed with a fraction of inspired oxygen (FiO₂) of 75%. However, as peripheral oxygen saturation (SpO₂) decreased to the low 90 s, FiO₂ was increased to 100% and maintained thereafter. Anesthesia was maintained with desflurane at 6% and remifentanil at 100–700 µg/h. Surgical fixation of the clavicle fracture was performed in the beach-chair position. The operation was uneventful (77 min; anesthesia 155 min), without severe desaturation or hemodynamic instability (Fig. 2).

**Fig. 3:**
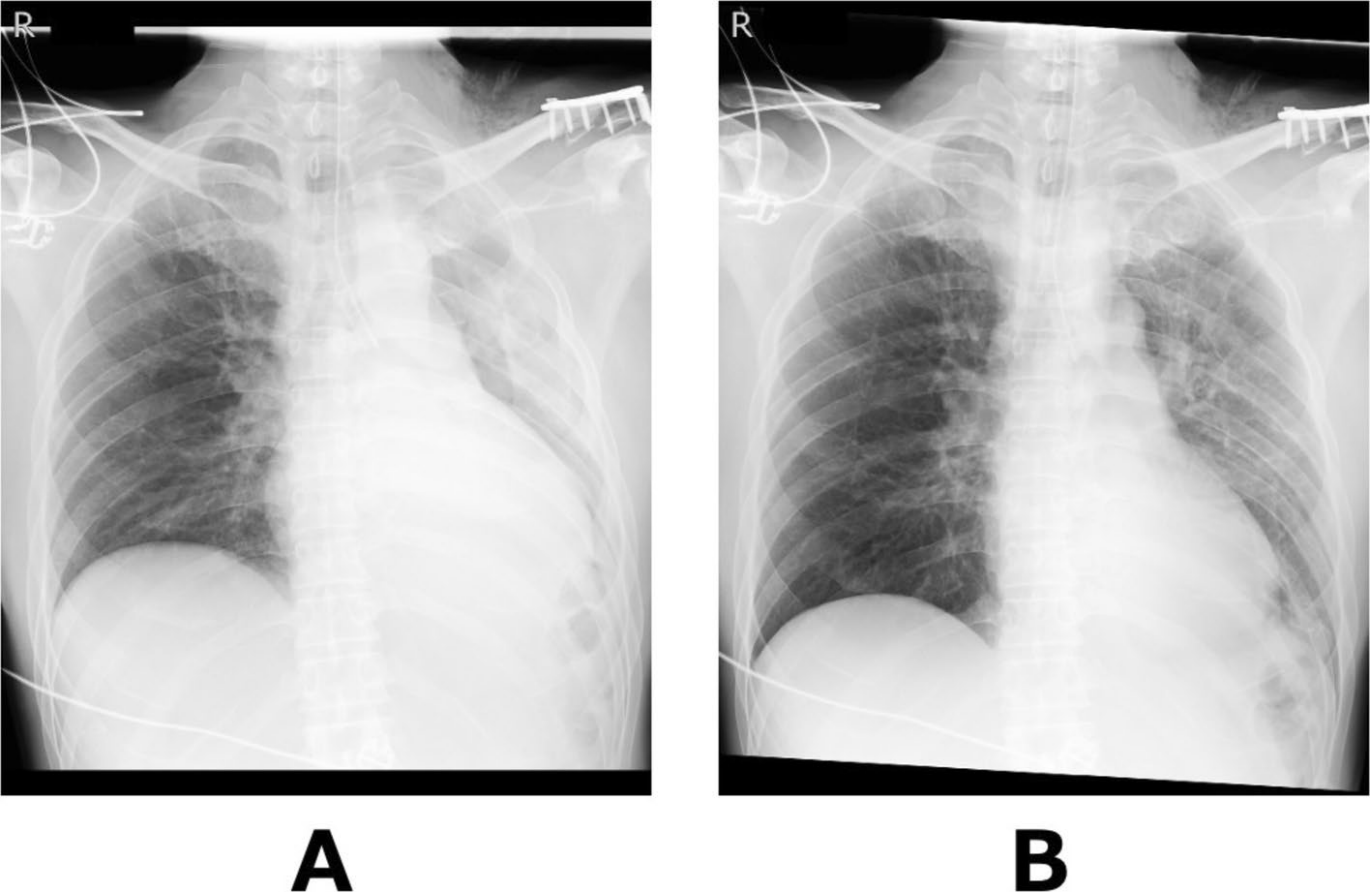
**A** Chest radiography while OLV was still in place showed decreased inflation of the left lung. **B** Left lung inflation improved after about one minute of gentle manual ventilation

**Fig. 4:**
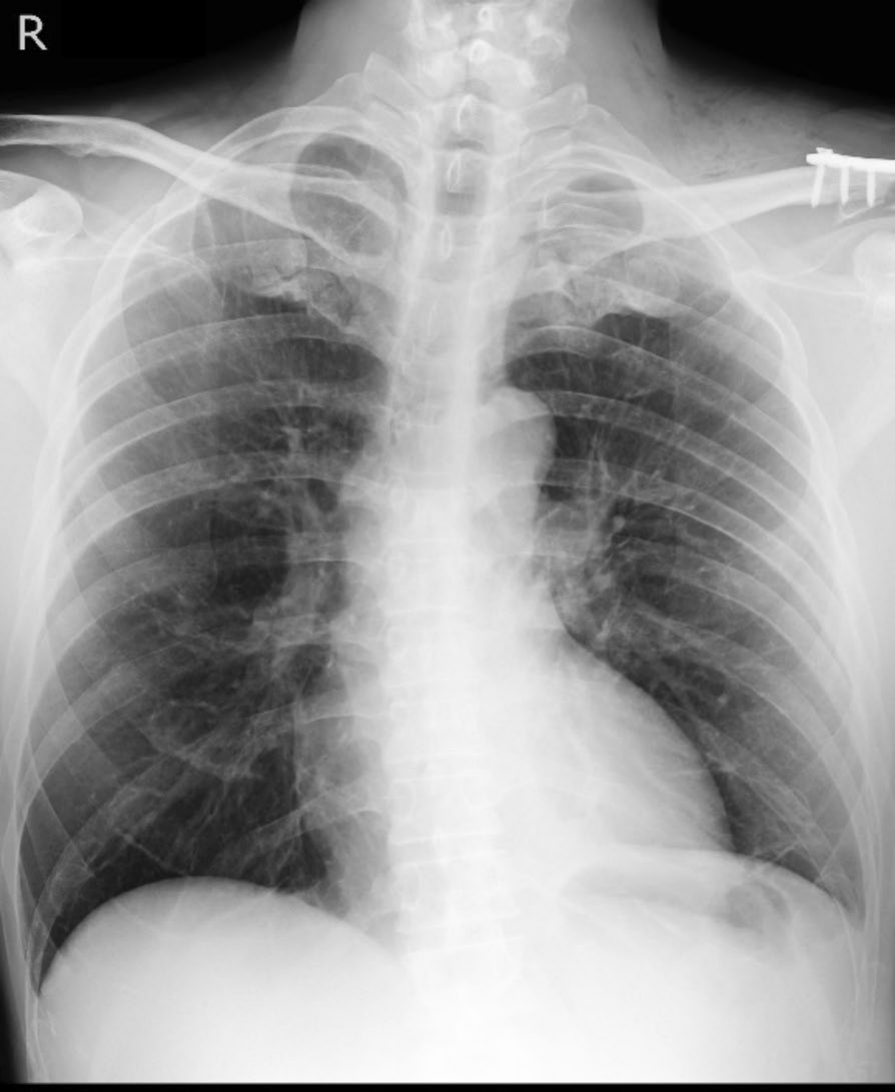
Chest radiography on postoperative day 1 showing further improvement of left lung inflation

Postoperatively, chest radiography performed while OLV was still in place showed decreased inflation of the left lung (Fig. 3A). Gentle manual ventilation was performed for approximately 1 min at airway pressures of 10–15 cmH₂O, and a follow-up chest radiograph confirmed re-expansion of the left lung (Fig. 3B). The patient was then awakened and extubated. On postoperative day 1, chest radiography showed further improvement in left lung inflation and no evidence of pneumothorax (Fig. 4). The patient had no postoperative complications and was discharged home on postoperative day 2. The patient remained well during outpatient follow-up, with no evidence of pneumothorax recurrence or respiratory symptoms.

## Discussion

To minimize the risk of exacerbating the existing pneumothorax, we deliberately selected OLV to avoid applying PPV to the injured lung. Although alternative strategies—such as regional anesthesia alone or in combination with general anesthesia using a supraglottic airway—would have been reasonable, they carry the drawback of potentially limited airway access in the beach-chair position if an emergency such as tension pneumothorax arises. Therefore, we opted for tracheal intubation and OLV, which allowed for both airway control and lung isolation. The surgery proceeded uneventfully without pneumothorax progression. We were prepared to convert to bilateral ventilation if oxygenation became inadequate and to perform emergency chest drainage if needed. Neither intervention was ultimately necessary.

In patients with OPTX, the risk of worsening during general anesthesia with bilateral PPV—when prophylactic chest drainage is not performed—remains unknown. However, several reports from ward and intensive care unit settings may provide relevant insights. In a retrospective study of 602 patients with traumatic pneumothorax, Walker et al. reported that 10% of patients who received PPV without chest drainage eventually required chest tube placement during their clinical course [4]. Similarly, the prospective OPTICC study demonstrated that approximately 25% of OPTX patients in the observation group who initially received PPV without chest drainage later required chest tube placement [5]. These findings suggest that, in OPTX patients undergoing general anesthesia with bilateral PPV, the likelihood of intraoperative pneumothorax worsening requiring chest drainage is a clinically relevant risk that should not be overlooked. If prophylactic chest tube placement is not performed, alternative strategies—such as preserving spontaneous respiration or employing OLV to limit PPV on the affected side—should be considered.

Although OLV is a well-established technique for achieving lung isolation, particularly in thoracic anesthesia, it carries a known risk of hypoxemia. In our case, moderate hypoxemia developed during OLV, and FiO₂ had to be increased to 100% to maintain SpO₂ above 90%. Typically, OLV is performed in the lateral decubitus position, with the non-ventilated lung positioned uppermost and open to the atmosphere, allowing it to collapse. The combination of lung collapse and gravity-dependent redistribution of pulmonary blood flow reduces perfusion to the non-ventilated lung, thereby improving ventilation-perfusion matching and mitigating hypoxemia [6]. However, in the present case, the non-ventilated hemithorax was not open to air, and the lung did not collapse. Furthermore, the beach-chair position lacks the gravitational advantage of the lateral decubitus position in diverting blood flow away from the non-ventilated lung. These factors likely contributed to the observed hypoxemia during OLV.

In summary, this case illustrates that OLV without prophylactic chest drainage can be a feasible strategy for managing clavicle fracture surgery in patients with OPTX, provided that careful preparation is made for potential hypoxemia and pneumothorax progression. Compared to pulmonary surgery, greater attention may be required for hypoxemia during OLV in such cases, where lung collapse and gravity-dependent perfusion redistribution may not occur. This approach may be suitable for patients with stable, small pneumothoraces and preserved respiratory function, provided that emergent drainage is readily available. Conversely, it may be contraindicated when pneumothorax is large, worsening, or when emergency access is limited.

## Data Availability

Not applicable.

## Abbreviations

PPV: Positive pressure ventilation
CT: Computed tomography
OPTX: Occult pneumothorax
OLV: One-lung ventilation
FiO_2_: Fraction of inspired oxygen
SpO_2_: Peripheral oxygen saturation
